# Anaplastic thyroid carcinoma with diffuse thoracic skin metastasis: A case report

**DOI:** 10.3892/ol.2014.2036

**Published:** 2014-04-04

**Authors:** SERDAR ALTINAY, BETÜL TAŞ, AYNUR ÖZEN, PELIN ALTINOK SÜT

**Affiliations:** 1 Department of Pathology, Bağcılar Training and Research Hospital, Istanbul 34203, Turkey; 2 Department of Dermatology, Bağcılar Training and Research Hospital, Istanbul 34203, Turkey; 3 Department of Nuclear Medicine, Bağcılar Training and Research Hospital, Istanbul 34203, Turkey; 4 Department of Radiation Oncology, Bağcılar Training and Research Hospital, Istanbul 34203, Turkey

**Keywords:** thyroid, anaplastic carcinoma, skin metastasis, thoracic, paclitaxel

## Abstract

Anaplastic thyroid carcinoma is a significantly fatal endocrine neoplasm, with an average survival time of 4–12 months following diagnosis. The present study reports the case of a 57-year-old male patient who presented to the Bağcılar Training and Research Hospital (Istanbul, Turkey) due to swelling in the neck and difficulty swallowing. The jugular mass biopsy results were consistent with a diagnosis of anaplastic thyroid cancer. The patient was regarded to have advanced-stage subcarinal, paratracheal, aortopulmonary, trancheobronchial and mediastinal lymphadenopathies and exhibited a good prognosis following chemotherapy. However, the patient succumbed one month later due to the emergence of diffuse skin lesions. The histopathological and immunohistochemical assessment of the skin biopsy displayed the characteristics of the underlying thyroid carcinoma.

## Introduction

Anaplastic thyroid carcinoma (ATC) is one of the most aggressive endocrine tumors, with a high mortality rate. ATC accounts for only 1–2% of all thyroid cancer and the mean age at diagnosis is between 55 and 65 years (1–3). ATC is a rare tumor that may metastasize to the skin in the context of diffuse body metastases (4). By contrast, cutaneous metastasis from differentiated thyroid carcinoma is a rare manifestation of disseminated disease, while the most common site of thyroid carcinoma skin metastases is the scalp. Thoracic involvement is extremely rare. The metastatic deposits usually present as flesh coloured nodules that are tender, often itchy and which may ulcerate (3,4). Owing to the relative rarity of this tumour, few treatment modalites are available and the selection of treatment is largely made on anecdotal evidence (2,4). ATC has a relatively negative prognosis following diagnosis, with an average survival time of 4–12 months (1,2).

The current study describes a case of anaplastic thyroid cancer that presented with cervical lymph node metastasis and exhibited diffuse skin metastases over a number of areas, excluding the scalp, following treatment. The clinical, radiological and pathological findings are presented. The patient provided written informed consent.

## Case report

A 57-year-old male presented to the Bağcılar Training and Research Hospital (Istanbul, Turkey) due to difficulty swallowing and swelling in the neck. The patient indicated a previous diagnosis of goiter and had lost 3 kg of weight within the previous 2 months. Laboratory assessments demonstrated normal levels of free triiodothyronine, 2.38 pg/ml (ref, 2–4.4 pg/ml), free thyroxine, 1.16 ng/dl (ref, 0.9–1.7), and thyroglobulin, 7.25 ng/ml (ref, 3–40 ng/ml), and a lower than normal level of thyroid-stimulating hormone, 0.01 mU/l (ref, 0,35–5,5). The sedimentation level was 11 mm for 30 min (ref, 0–5 h), 33 mm for 1 h (ref, 0–20) and was high overall. All other values were within the normal ranges.

**Figure 1:**
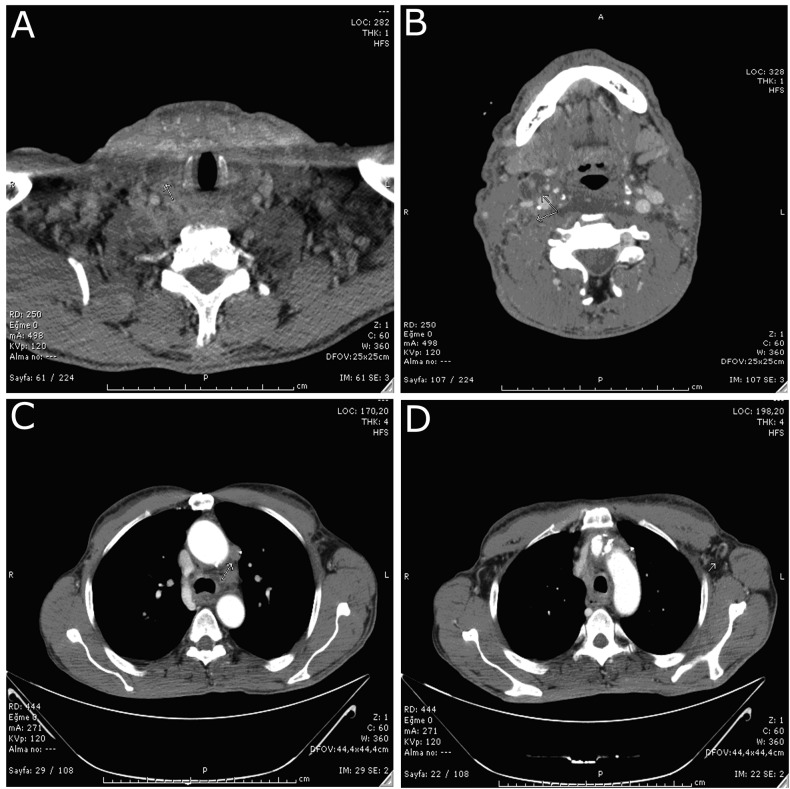
CT scan of the neck revealing a hypodense lesion in the (A) thyroid gland and (B) metastatic cervical lymphadenopathies. CT scan of the thorax showing a metastatic lymphadenopathy in the (C) aorticopulmoner window and (D) lymphadenopathy in the left axilla. CT, computed tomography.

The neck computed tomography (CT) scan performed in January 2012 exhibited a heterogeneous appearance, with an increase in the size of the thyroid gland and marked parenchyma. Lobular-contoured, centrally cystic-necrotic lymphadenopathies were observed in the two jugular chains, with those in the right jugular chain being more marked; the larger ones at a size of 2 cm and others having a conglomerate appearance. Subcarinal, right paratracheal, aortopulmonary, paraesophageal and left tracheobranchial mediastinal lymphadenopathies were detected during the thoracic CT, with the largest measuring 23 mm in diameter (Fig. 1). Proximal esophageal constriction was observed in association with extrinsic pressure during the gastroscopy, which was applied due to the difficulty in swallowing.

**Figure 2:**
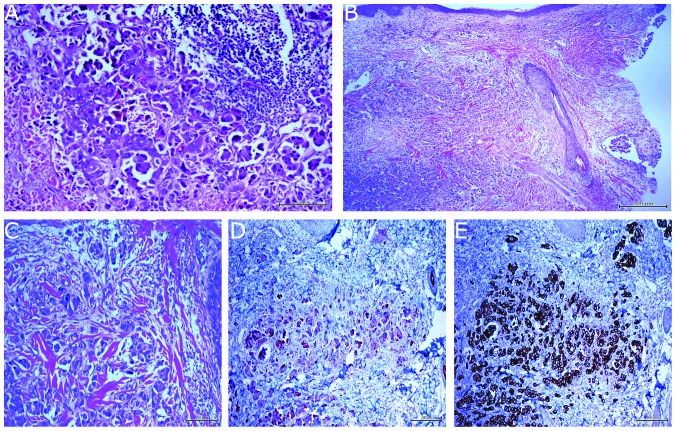
Histological features. (A) Anaplastic cells with bizarre nuclei can be observed adjacent to the lymphoid tissue (HE; magnification, ×20). (B) Similar tumor cells are present in the skin biopsy (HE; magnification, ×4). (C) The tumor is composed of pleomorphic, epithelioid and sarcomatoid cells at a magnification of ×20. Immunohistochemical features show strong immunoreactivity with (D) galectin-3 and (E) CK-7 in the tumor cells (magnification, ×10). HE, hematoxylin and eosin; CK, cytokeratin.

A fine-needle aspiration biopsy performed on a 20-mm lymphadenopathy in the right jugular chain resulted in a sample formed of large pleomorphic atypical epithelioid cells forming flaccid clusters. The histopathological characteristics of the Tru-Cut biopsy (Pro-Mag™ Ultra; PBN Medicals Denmark A/S, Stenløse, Denmark) material, which was retrieved from the same mass, was a match to the cytological aspirate (Fig. 2). The tumor cells showed positive immunoreactivity for cytokeratin 7 (CK7) and galectin-3 in the immunohistochemical staining, while exhibiting negative immunoreactivity for CK20, thyroid transcription factor-1 (TTF-1) and human melanoma black-45. In light of all these findings, the patient was diagnosed with a metastatic anaplastic thyroid carcinoma.

Due to the extensive nature of the anaplastic histology and lesions upon positron emission tomography (PET)/CT, the patient was regarded to have advanced head-neck cancer, and three cycles of doxorubicin (50 mg/m^2^) plus cisplatin (40 mg/m^2^) for three weeks were subsequently administered.

**Figure 3:**
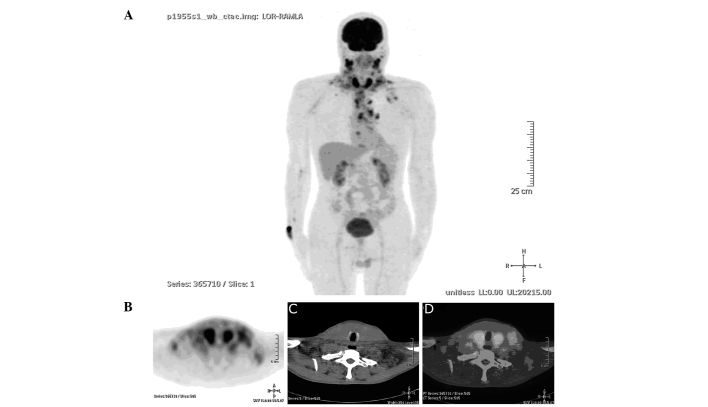
Post-treatment PET/CT maximum intensity projection images. (A) Diffuse-fluorodeoxyglucose uptake in the thyroid lobe and hypermetabolic lymph nodes in the mediastinal and neck regions. (B) PET, (C) CT and (D) fusion images of the axial-cross section of the thyroid. PET/CT, positron emission tomography/computed tomography.

^18^F-fluorodeoxyglucose (FDG; 12.33 mCi) was administered to the patient. PET/CT was subsequently performed when the blood glucose level was 107 mg/dl following 8 h of fasting for the assessment of the response to treatment after chemotherapy and planning radiotherapy. Extremely intense FDG involvement was observed in the two lobes of the thyroid, and a partial metabolic response of metastatic character was observed in the hypermetabolic lymphadenopathy in the neck, mediastinum and left axillary fossa (Fig. 3).

Six cycles of chemotherapy (50 mg/m^2^ doxorubicin and 40 mg/m^2^ cisplatin for three weeks) were administered upon the detection of anaplastic thyroid carcinoma metastasis in the axillary lymphadenopathy material extracted in April 2012. Palliative neck radiotherapy was applied up to a total of 70 gy in 20 fractions.

**Figure 4:**
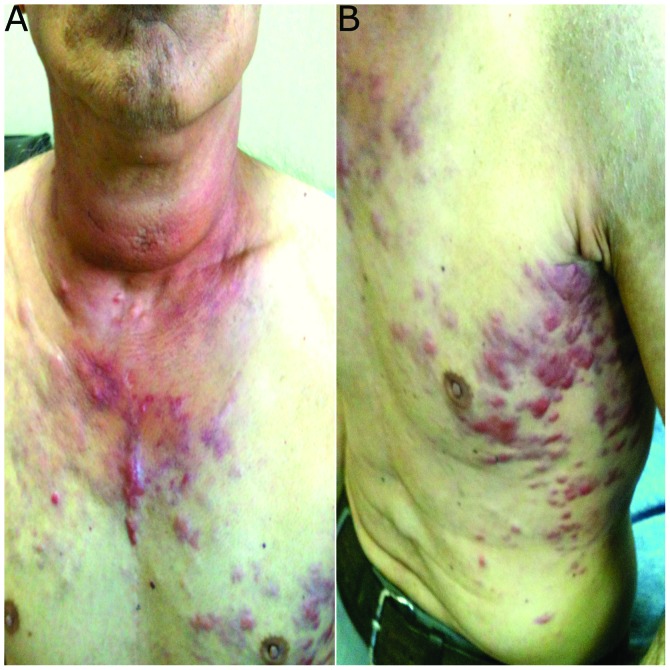
Diffuse, round to oval-shaped, purple to violet-colored skin lesions in the presternal and left infraaxillary regions. The lesions cover the (A) left front-lateral part of the thorax and extend to the (B) left infracostal border.

Upon the completion of the treatment, diffuse, painless, round to oval-shaped, purple to violet-colored skin lesions that were fixed beneath the skin were noticed and began to exhibit induration. The lesions appeared in the presternal and left infraaxillary regions in the first month, then covered the left front-lateral part of the thorax and extended to the left infracostal border. The lesions were 3–4 cm in size and occasionally formed plaques by merging (Fig. 4). Although the scalp is the most common site of involvement (4), no lesions were identified on the scalp of the current patient. The histopathological and immunohistochemical findings of the skin biopsy confirmed anaplastic thyroid carcinoma metastasis (Fig. 2). Consequently, the patient, who had received 2 rounds of paclitaxel (2.5 mg/kg) plus carboplatin (360 mg/m^2^) therapy, succumbed to severe respiratory failure 1 month after the development of the metastatic skin lesions.

## Discussion

Systemic metastases of thyroid cancers emerge during the disease in 75% of patients and occur most frequently in the lungs (80%), the bones (6–15%) and the brain (5–13%) (3). To date, ~50 cases have been reported in association with primary thyroid cancer. A study by Dahl *et al* (4) found that the most frequent thyroid cancer to give rise to skin metastases was papillary carcinoma, accounting for 41% of the cases, followed by follicular carcinoma at 28%, and then anaplastic carcinoma and medullary carcinoma each constituting 15% of the cases. By contrast, Koller *et al* (5) reported that follicular carcinoma has a greater preponderance for cutaneous metastases than papillary carcinoma. Furthermore, specific studies state that it is mostly papillary carcinomas that result in metastasis (3,4). Anaplastic thyroid carcinoma is a rare aggressive tumor that can lead to skin metastasis (6).

Skin metastases usually occur in the setting of disseminated neoplastic disease, and the presence of cutaneous metastases portends a poor prognosis. Skin metastasis typically presents as slowly growing, solitary or multiple, erythematous, flesh-colored, violaceous or blue-colored papules or nodules, usually on the scalp, face or neck (5,6). Scalp involvement was observed in 2/3 of patients in the study by Dahl *et al* (4). Thoracic skin involvement as observed in the present case is extremely rare. Violaceous, multiple papillary-nodular eruptions appeared with sudden onset and were diffused over the body, with different localizations compared with previous studies. The most frequently involved sites remained intact. However, the potential of skin metastasis, considered upon the diagnosis of primary carcinoma, was confirmed with histopathological assessment and immunohistochemical staining. Although rarely observed at the onset of the disease, skin metastases are generally observed in the advanced phases of neoplasia and are indicators of a bad prognosis (5,6). In the present case, the skin lesions appeared in the final phase in a similar way to those in previous studies, and the patient succumbed after a short 1-month period subsequent to their emergence (4–6). This period was shorter compared with the average survival period of 19 months (4), which has previously been indicated for post-cutaneous metastasis in the literature. Thyroid cancers with skin involvement may easily be confused with primary skin adnexial tumors. TTF-1 is considered to be beneficial for distinguishing carcinomas with pulmonary and thyroid immunohistochemistry from other primary cancers, mesotheliomas and primary cutaneous Merkel cell carcinomas (7).

The widest possible surgical approach may be applied in resectable tumors; those with an absence of unilateral extrathyroidal invasion at a diameter of <5 cm or those without cervical lymph node involvement (8). Considering the radiological findings, the patient of the present case had an advanced anaplastic carcinoma and had no surgical options.

It has been reported that the combination of doxorubicin and cisplatin is more effective compared with the single use of doxorubicin in complete response (9). Skin lesions occurred pursuant to the doxorubicin plus cisplatin therapy administered initially to the present study patient, and paclitaxel therapy was initiated upon the histopathological detection of ATC metastasis. Although this was a promising option, as a response rate of 53% has been observed previously (10), the patient did not benefit from the treatment. Although the average survival rate following cutaneous metastasis has been reported to be 19 months in the literature (4), the present patient succumbed to respiratory failure one month after the occurrence of skin metastasis.

In conclusion, the present case was one of anaplastic cancer, with skin metastasis emerging following treatment and diffuse thoracic involvement, excluding the scalp, 10 months after the diagnosis. The present study demonstrated that the possibility of metastatic thyroid cancer should be considered in solid, multiple violaceous, blue-colored thoracic skin lesions, and that metastatic thyroid carcinoma involving the skin can be mistaken for primary skin tumors. Immunohistochemical stains should be used in addition to the histopathological examination for the differential diagnosis. External beam radiotherapy may be used as an option for those patients who are not suitable for surgery or full tumor removal.
